# Robotic low anterior resection plus transanal natural orifice specimen extraction in a patient with situs inversus totalis

**DOI:** 10.1186/s12893-018-0394-3

**Published:** 2018-08-20

**Authors:** Beibei Cui, Sanlin Lei, Kuijie Liu, Hongliang Yao

**Affiliations:** Department of Gastrointestinal Surgery, The Second Xiangya Hospital, Central South University, No.139 Middle Renmin Road, Changsha, Hunan 410011 People’s Republic of China

**Keywords:** LAR, NOSE, SIT, Rectal cancer, Case report

## Abstract

**Background:**

Situs inversus totalis (SIT) refers to an unusual condition involving reversal of abdominal and thoracic viscera, with an incidence rate of 1/5000–20,000 adults. Minimally invasive surgeries for SIT patients are technically challenging, while the surgical experience for SIT patients is quite limited.

**Case presentation:**

A 61-year-old man, previously diagnosed as SIT, came to our hospital for 6 months history of hematochezia and altered bowel habit. A diagnosis of rectal cancer was made in view of colonoscopic biopsy which confirmed an irregular circumferential lump of well differentiated adenocarcinoma at 10 cm from the anal verge. The computed tomography contrast-enhanced (thorax + abdomen + pelvis) scan revealed a total transposition of abdominal and thoracic organs and an enhanced eccentric mass of rectal but with no evidence of distant metastasis. Robotic low anterior resection (LAR) plus transanal natural orifice specimen extraction (NOSE) was performed after obtaining informed consent. The procedure was performed successfully and the patient convalesced nicely without any complications. The postoperative pathological diagnosis revealed a 4x4x0.6 cm^3^ moderately differentiated adenocarcinoma and circumferential clearance.

**Conclusions:**

Robotic LAR plus transanal NOSE for rectal cancer patients with SIT can be performed safely and may be an effective approach in contrast to open or laparoscopic approach, despite the unconventional anatomy.

## Background

SIT refers to a rare congenital abnormality with an incidence rate of 1/5000–20,000 adults characterized by the transposition of abdominal and thoracic viscera, like a mirror image [1]. Due to the uncommon anatomy and the exact mirror image of the usual technique, surgical procedures for SIT patients are considered more difficult, especially in minimally invasive surgeries [2]. Robotic rectal surgery is currently a novel procedure for rectal cancers. Transanal NOSE is a novel technique to remove the specimen from the abdominal cavity through the anus instead of an additional incision following laparoscopic or robotic colorectal surgery [3]. To the best of our knowledge, there has been no case described in the literature that combined robotic LAR with transanal NOSE for SIT.

## Case presentation

**Fig. 1:**
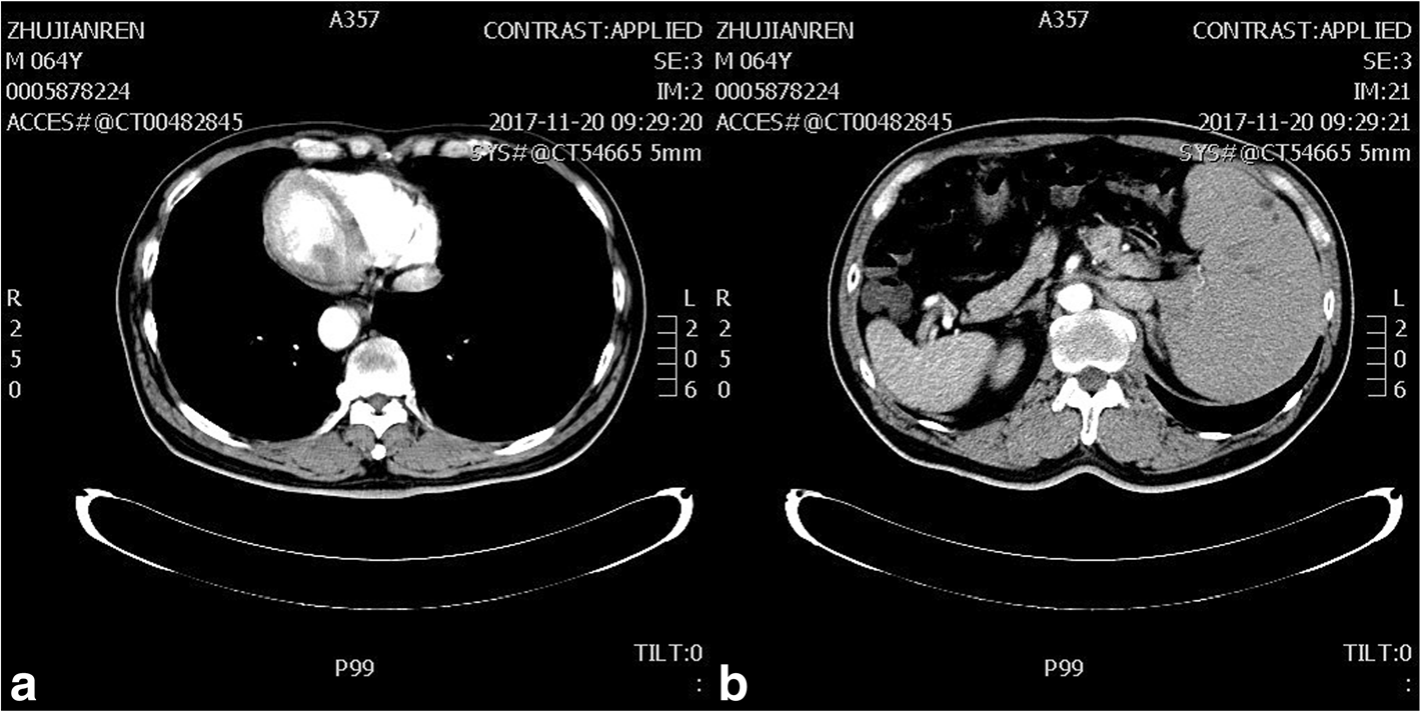
The preoperative computed tomography scan showed a complete transposition of the thoracic and abdominal viscera. **a** Mirror-image dextrocardia. **b** The liver was located on the left side of the abdomen

**Fig. 2:**
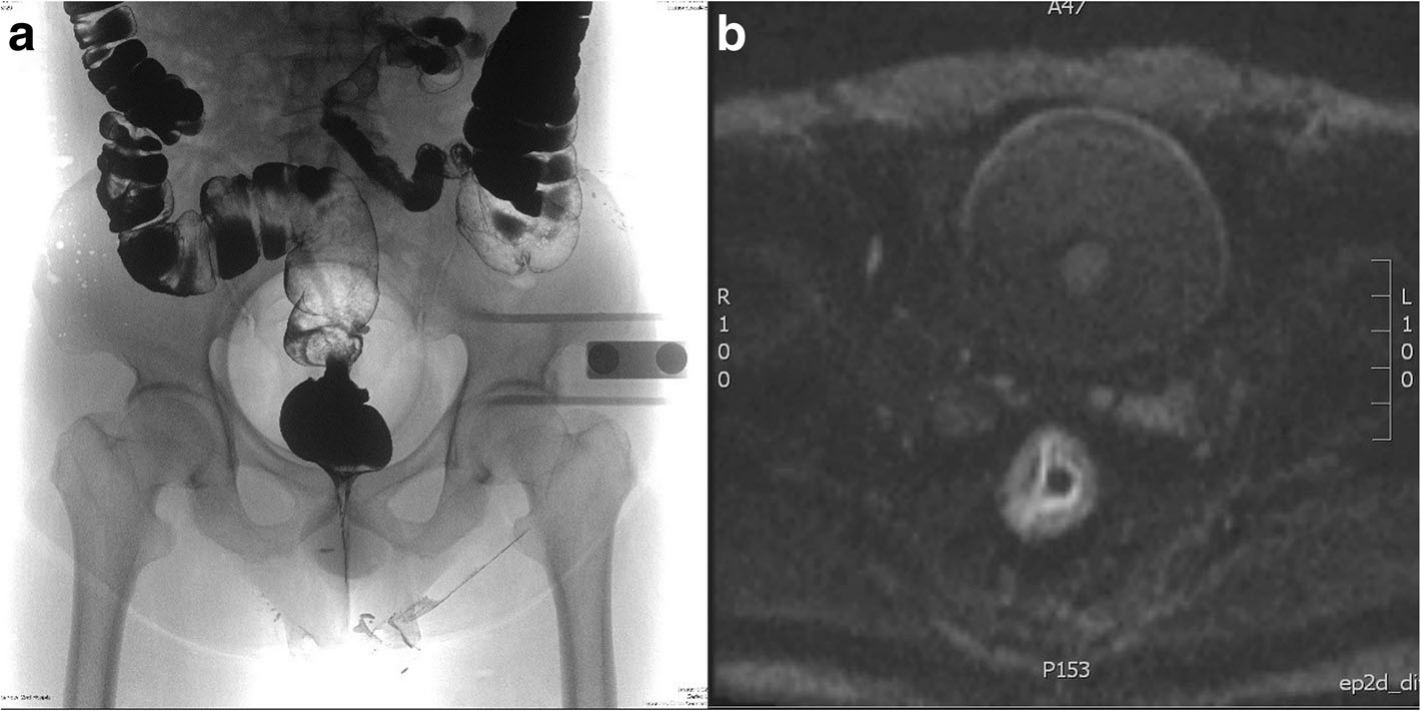
**a** The double-contrast barium enema showed an irregular rectal stenosis, nodulous filling defect and stiffness of involved rectal wall with destruction of mucosa. **b** The magnetic resonance imaging scan revealed the tumor invaded through the muscularis propria and the serosa was suspiciously involved, while at least 2 enlarged perirectal lymph node was found

A 61-year-old man, previously diagnosed as SIT, came to our hospital for 6 months history of hematochezia and altered bowel habit. A diagnosis of rectal cancer was made in view of colonoscopic biopsy which confirmed an irregular circumferential lump of well differentiated adenocarcinoma at 10 cm from the anal verge. And the preoperative chest X-ray image and computed tomography scan revealed a total reversal of abdominal and thoracic organs, proving SIT (Fig. 1). The double-contrast barium enema revealed an irregular rectal stenosis, nodulous filling defect and stiffness of involved rectal wall with destruction of mucosa. The magnetic resonance imaging showed the lump invaded through the muscularis propria and the serosa was suspiciously involved, while at least 2 enlarged perirectal lymph nodes were found, while the computed tomography (thorax + abdomen + pelvis) scan showed no distal metastasis (Fig. 2). The remaining of the routine blood results were not abnormal, save a slightly decreased haemoglobin and albumin level, 12.9 g/dL and 3.8 g/dL. After obtaining informed consent, Robotic LAR with transanal NOSE was performed.

Preoperative mechanical bowel preparation was carried out with polyethylene glycol electrolytes powder. Streptomycin and metronidazole were given as antibiotic prophylaxis. The operation was performed under general narcosis while the position of the patient was adjusted to a modified lithotomy position. The patient was placed in the Trendelenburg position at 30 degree and 10-degree tilted right-side-up.

**Fig. 3:**
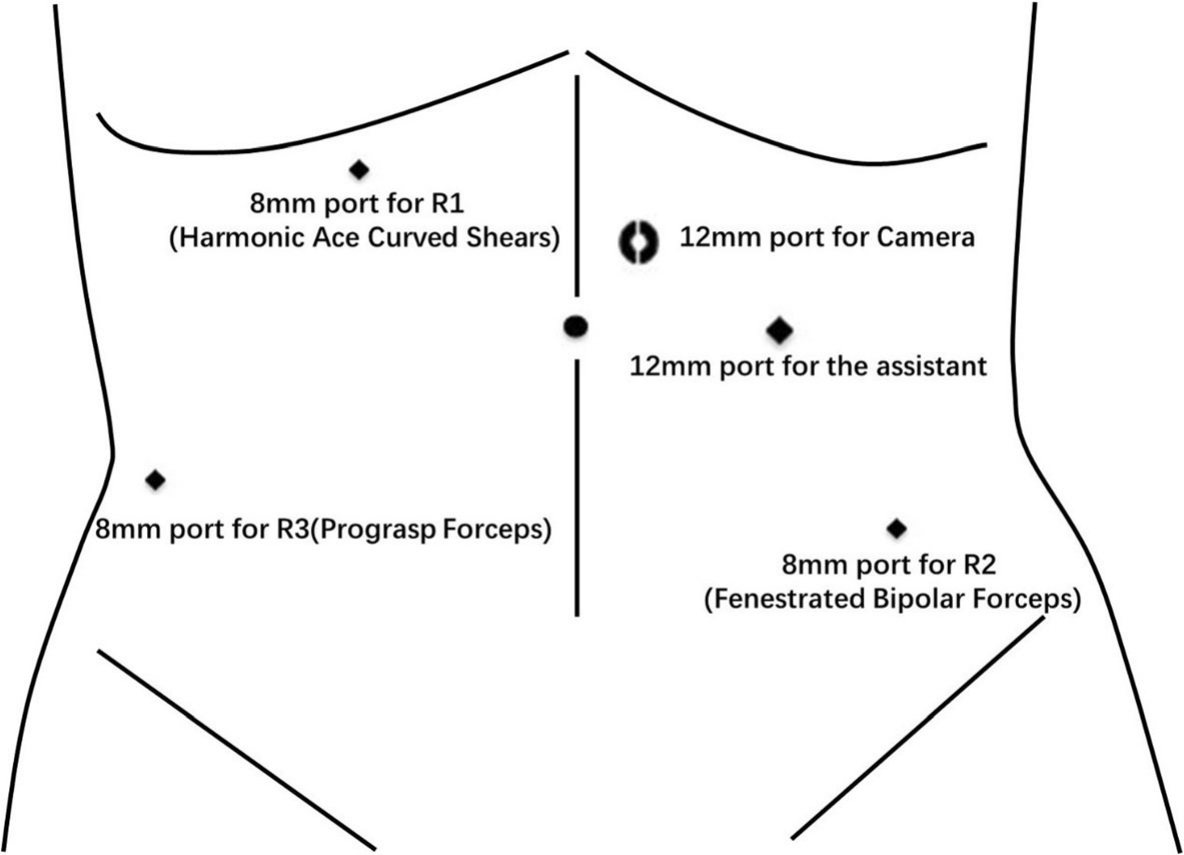
Port placement for robotic LAR

A 5-port method was adopted: two 12-mm ports for the camera and the assistant respectively, three 8-mm robotic ports. Pneumoperitoneum was established with the Veress needle approach under direct vision and a 12-mm camera port was inserted in 2 cm superior and right lateral to the umbilicus. Laparoscopic exploration of the abdominal cavity confirmed a total transposition of the abdominal organs and no abnormality from the descending to the sigmoid. The mass, in a size of 5*4*4cm^3^ and locating 2 cm above peritoneal reflection, suspiciously invaded the serosa without significant perirectal lymph node metastasis. The 8-mm port for robotic arm 1 was inserted in the right mid-clavicular line, 4 cm superior to the umbilicus, while the 8-mm port for robotic arm 3 was inserted in the right anterior axillary line, 4 cm inferior to the umbilicus. The third 8-mm trocar for robotic arm 2 was placed in the left lower quadrant (LLQ) that is one-third of the distance from the anterior superior iliac spine to the umbilicus. Harmonic ace curved shears, Prograsp forceps and Fenestrated bipolar forceps were respectively installed in the robotic arms 1, 3, and 2. The 12-mm assistant port was inserted in the right mid-clavicular line, parallel to the umbilicus. A 30-degree camera lens was adopted (Fig. 3).

After finishing installation of the various ports, the small intestine was swept to the LUQ to expose the right lower quadrant. The procedure started from incising the peritoneum at the inferior mesenteric artery root by means of Harmonic ace curved shears. The inferior mesenteric vessels were denuded, clipped, and divided. Then the peritoneum of the sigmoid mesentery was incised over the sacral promontory just to the left of midline. With cephalic traction and countertraction to the sigmoid mesentery provided by Fenestrated bipolar forceps and Prograsp forceps, a vascular free plane between the mesentery and the retroperitoneum was developed. After identifying and safeguarding the right ureter, the plane was developed towards the caudal and lateral direction further. Then it was possible to mobilize the descending colon mesentery further. The whole descending colon and sigmoid were mobilized well from the lateral pelvic. Splenic flexure mobilization was also performed.

The next step of the procedure was mesorectal dissection. The dissection was started from posterior to the rectum, at the level of the sacral promontory, and the peritoneum was incised along both sides of the rectum down to and around the anterior peritoneal reflection. The proximal line of resection was identified at 14 cm to the superior margin of the tumor, and the proximal end of the specimen was ligated with a special plastic seal, while the distal end of the specimen was ligated at 4 cm to the inferior margin of the tumor. Then the proximal colon and the distal colon were successively transected and divided using Harmonic ace curved shears.

For specimen extraction, we used a specially designed bag with an adjustable loop of string which could close the bag. The bag was introduced through the anus, and the specimen was deposited and closed inside the bag. Following carefully dilating the anus until two fingers could be easily reached, the specimen were then grasped and slowly “snaked” out of the bag through the anus. The bag was then removed.

**Fig. 4:**
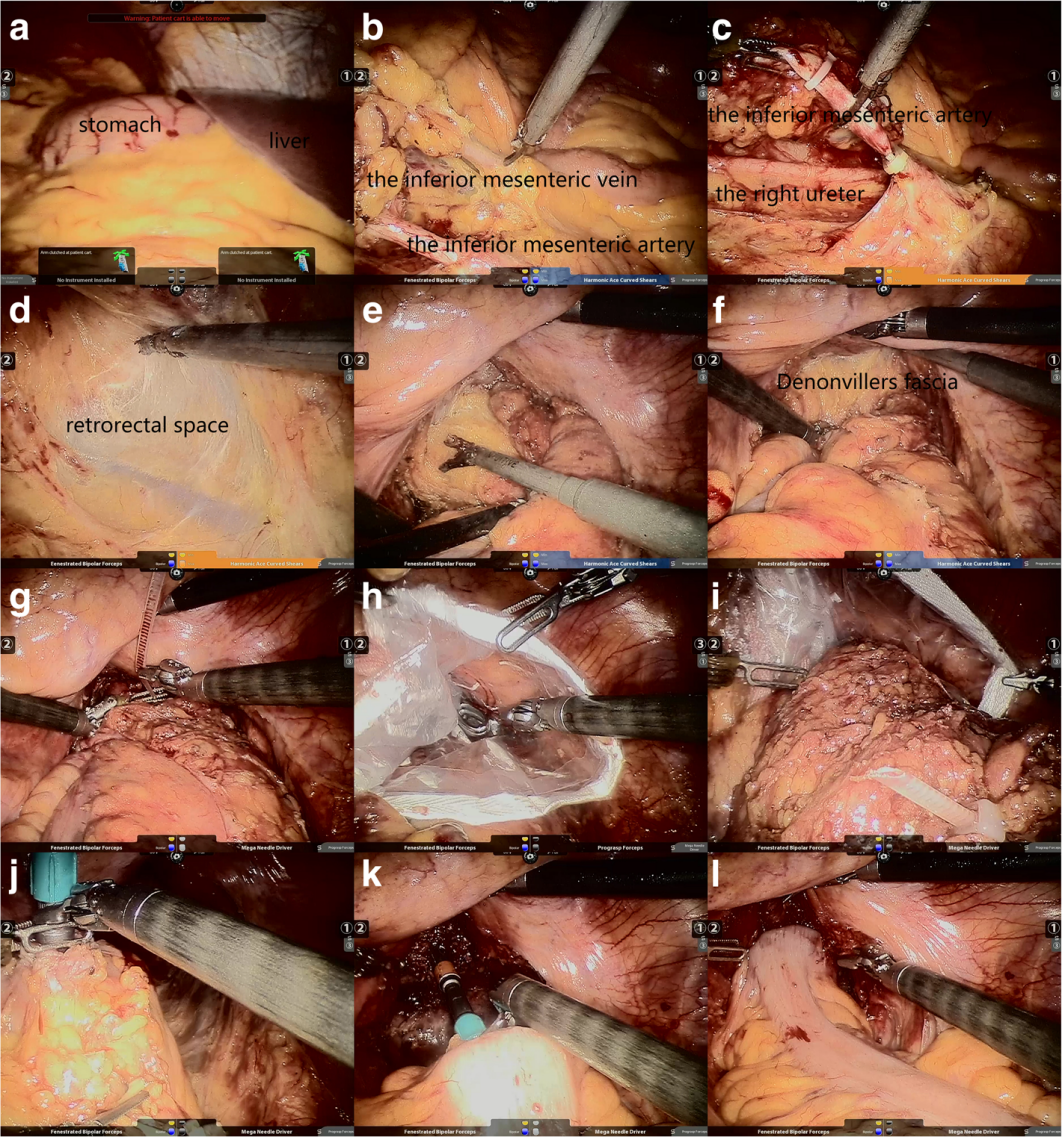
**a** A complete transposition of the abdominal viscera revealed by laparoscopic exploration. **b** Denudation and division of the inferior mesenteric vessels. **c** Recognition and preservation of the right ureter. **d-f** Dissection of retrorectal space and Denonvillers fascia for mesorectal excision. **g-i** Transanal NOSE j-l. Intraperitoneal anastomosis

The aperture of the proximal colon had been previously estimated and an appropriate stapler was chosen. According to our experience, 31 mm EEA circular stapler allowed the anvil passing and resulted in a very sufficient lumen. The anvil was introduced into the abdominal cavity through the anus and was fixed in the proximal margin of the previously opened proximal colon by purse-string suture, while the redundant tissue around the anvil was cut off to ensure a full exposure of the tissue with staples. Following introducing the CEEA stapler through the anus, the second assistant slowly extruded the spike through the center of the rectal stump and performed a colorectal anastomosis with double-checking the amount of tension. We filled the pelvis with distilled water and injected air in the reconstruct bowel to ensure any leak being repaired in time by means of intracorporeal sutures (Fig. 4).

After confirming anastomotic integrity, the whole abdominal cavity, and especially the trocars were watered with distilled water, bromogeramine solution and saline solution successively, and the peritoneal cavity was suctioned dry. And a closed suction drain was placed into the pelvic cavity. After removing the trocars, all port sites were immediately closed with sutures in a subcuticular fashion.

**Fig. 5:**
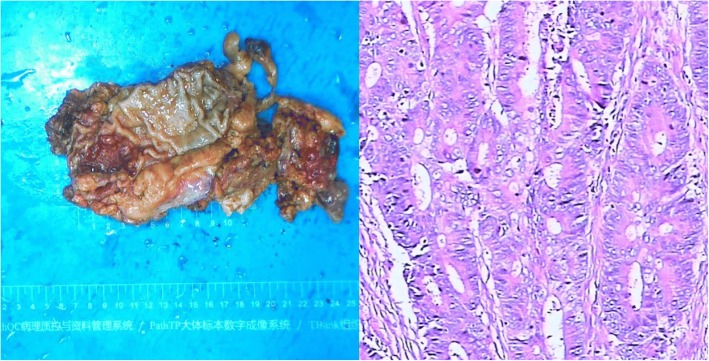
The postoperative pathological diagnosis revealed a 4x4x0.6 cm^3^ moderately differentiated adenocarcinoma invading the muscularis propria without perirectal lymph node metastasis (T2 N0) with a 1 cm distal and circumferential clearance

In this case, the total operative time was 210 min, while the docking time and the console time was 25 min and 185 min respectively. Estimated blood loss was less than 50 mL. The first flatus and liquid diet happened on the second day after the operation, while solid diet the fifth day. The patient convalesced nicely without any complications and was discharged on the seventh day after the surgery. The postoperative pathological diagnosis revealed a 4x4x0.6 cm^3^ moderately differentiated adenocarcinoma (T2 N0) with a 1 cm distal and free microscopic circumferential margin. The number of lymph nodes harvested is 13, lymph node retrieval was performed by pathology technicians (Fig. 5).

## Discussion and conclusion

SIT refers to a mirror image reversal of abdominal and thoracic viscera. The exact mechanism that controls the reversal of abdominal and thoracic viscera is unknown. It is believed that the rotation of the heart initiates the process of rotation and migration of the abdominal organs. Researchers have also found a protein named “Sonic hedgehog” is associated with organ rotation and migration. If the “Sonic hedgehog” protein is secreted on the right side, the heart loops to the left, resulting in situs inversus [4, 5]. We have no previous experience in robotic rectal surgery for SIT patient, so an electronic search of Pubmed and Google Scholar was performed, using the terms “situs inversus” and “rectal cancer”. Owing to its rarity, only five English articles on minimally invasive surgery for rectal cancer patients with SIT were retrieved, so the surgical experience we can learn from is very limited [2, 6–9].

Although there are several benefits about laparoscopic LAR for patients: less postoperative pain, better cosmesis, earlier recovery of intestinal function, and shorter hospital stay, the drawbacks inherent in this approach, such as an unstable 2-D (two-dimensional) view, hand tremor amplification, uncomfortable ergonomic positions for surgeons, restricted ranges of instrument movement within the confines of the pelvic space, mean that this procedure is technically challenging. Robotic approach provides some advantages over conventional laparoscopic approach that potentially overcome above mentioned drawbacks, including three-dimensional vision, less fatigue, tremor filtering and seven degrees of wrist-like motion [10, 11]. These technical advantages seem to be especially helpful in rectal cancer surgeries, particularly in the performance of total mesorectal excision (TME) within the narrow pelvic region.

NOSE has been developed as a novel method to remove the resected specimen from the abdominal cavity through a natural orifice instead of an additional incision after laparoscopic or robotic colorectal operations. The superiority related to NOSE includes avoiding additional abdominal wall trauma, reducing pain, and shorting hospital stay [3]. Although it has been applied to laparoscopic colorectal surgeries, challenge in performing an anastomosis laparoscopically is the key obstacle. Technical innovations in the robotic approach may aid in overcoming the technical drawbacks of the laparoscopic NOSE procedure for rectal cancer surgery. NOSE in robotic colorectal surgeries mean real minimal invasive procedures [12, 13].

To the best of our knowledge, there has been no case described in the literature that combined robotic LAR with transanal NOSE for SIT. In this paper, we present our surgical technique and short-term outcomes of robotic LAR plus transanal NOSE in a SIT patient. Based on our limited previous experience, we have not observed any serious malfunction of the neorectal, transanal NOSE in robotic rectal cancer resection is feasible and safe without sacrificing oncological efficacy.

Robotic rectal surgery is a novel technique, but the principles of the procedure is just like the laparoscopic. Sufficient preoperative anatomical consideration and procedure planning is vitally important in the presence of SIT. In the operation, the surgeon needs to adapt to the abnormal anatomy, while the robotic port locations, robotic arm positions, and assistant and nursing positions need to be modified. The operation was performed by Prof. Yao, an experienced surgeon who has performed more than 260 robotic colorectal surgeries. In this case, the docking time was 25 min while console time 185 min. Blood loss was < 50 mL. The surgeon’s right hand controlled a Harmonic ace curved shears to dissect, while Prograsp forceps and Fenestrated bipolar forceps respectively controlled by the right hand and the left hand for retraction. Although some degree of maladjustment was experienced at the beginning of surgery, this was easily overcome by the technological superiority provided by the robotic system. The superior three-dimensional vision enabled definite recognition of the important structures, like the right ureter, even though the unconventional anatomy. The seven degrees of wrist-like motion and the tremor filtering function also enhanced precise and complex performance in quite confined spaces of the pelvis. These advantages of the da Vinci system make tissue dissection relatively easy and safe.

Owing to unique advantages of the da Vinci system, robotic LAR plus transanal NOSE for rectal cancer patients with SIT can be performed safely and may be an effective approach in contrast to open or laparoscopic approach, despite the unconventional anatomy.

## Abbreviations

LAR: Low anterior resection
NOSE: Natural orifice specimen extraction
SIT: Situs inversus totalis
TME: Total mesorectal excision
